# Selective Influences of Precision and Power Grips on Speech Categorization

**DOI:** 10.1371/journal.pone.0151688

**Published:** 2016-03-15

**Authors:** Mikko Tiainen, Kaisa Tiippana, Martti Vainio, Tarja Peromaa, Naeem Komeilipoor, Lari Vainio

**Affiliations:** 1 Division of Cognitive and Neuropsychology, Institute of Behavioural Sciences, University of Helsinki, Helsinki, Finland; 2 Phonetics and Speech Synthesis Research Group, Institute of Behavioural Sciences, University of Helsinki, Helsinki, Finland; Goldsmiths, University of London, UK, UNITED KINGDOM

## Abstract

Recent studies have shown that articulatory gestures are systematically associated with specific manual grip actions. Here we show that executing such actions can influence performance on a speech-categorization task. Participants watched and/or listened to speech stimuli while executing either a power or a precision grip. Grip performance influenced the syllable categorization by increasing the proportion of responses of the syllable congruent with the executed grip (power grip—[ke] and precision grip—[te]). Two follow-up experiments indicated that the effect was based on action-induced bias in selecting the syllable.

## Introduction

Perception-action theories (e.g., [1]) are based on the assumption that perceptual and motor-planning processes share a common system in which perception may influence motor processes, but motor processes could also have an influence on perceptual processes. In line with these theories, studies have shown, for example, that the size of a viewed object automatically activates the grasp motor program that is congruent with the size (e.g., small object-precision grasp) [2], and the processing of an object is improved if its size is congruent with the grip type that is prepared prior to the onset of the stimuli [3, 4]. The current study investigates these action-perception issues in the context of speech, addressing the question of whether manual grasp performance can influence the categorization of speech stimuli. The motor theory of speech perception is perhaps one of the most widely known accounts of how perception might interact with motor processes. In short, the theory posits that speech is perceived by recruiting the motor networks needed to produce speech [5, 6]. In line with this view, and perhaps of most relevance for the current study, it has also been shown that the activation of articulatory representations may also have an impact on speech categorization. Research findings indicate that the transcranial magnetic stimulation (TMS) of language-production areas influences syllable categorization [7–9]. Other evidence supporting the theory includes reported increased excitability of tongue muscles among subjects listening to speech sounds that require specific tongue movements [10]. It has also been found that a participant’s own silent overt articulation affects speech categorization [11–13].

Speech, in general, could be viewed in the wider context of gestures, there being tight interactions between articulatory and manual gestures. In fact, some influential theories of language evolution posit that spoken communication evolved from, or co-evolved with, gestural communication (e.g., [14, 15]). It could be expected in accordance with these theories that there is still a link between manual gestures and speech. In this connection, it has been found that grasping with the hand, or observing objects of different sizes being grasped, influences simultaneous articulations such that, for example, grasping larger objects results in larger mouth apertures during articulation [16–18].

Our group has recently identified specific connections between certain articulations and manual grips [19]. For example, pronouncing the syllable [ke] was associated with faster reactions with a power grip, which is used to grasp larger objects (e.g., an apple) by pressing the object between the fingers and the palm of the hand. In contrast, the syllable [te] was associated with faster reactions with a precision grip, which is used to grasp small objects (e.g., a grape) with the thumb and the index finger. These associations, we propose, demonstrate that certain articulatory gestures are programmed in a motor network, which partially overlaps with grasp motor representations. More precisely, the body of the tongue is used to block the airflow at the soft palate to produce [k], whereas the tip is used at the alveolar ridge to produce [t]. We suggest that articulations involving the body of the tongue could be thought of as articulatory equivalents of whole hand movements, in other words a power grip. On the other hand, articulations mainly involving the tip of the tongue could be thought of as analogous to hand movements using the tips of the fingers, like a precision grip [20]. This interpretation is in line with theories of mouth-hand mimicry according to which some articulations may be ‘synkinetic’ mimes of hand gestures (e.g., [21, 22]). Listening to syllables that are congruent or incongruent with the grip required for the response may also trigger the link between grip type and articulatory gestures, suggesting that overt articulation is not necessary to observe the effect [20].

The evidence discussed above shows the influence of perceived manual actions on articulation (e.g., [17]), and of perceived speech on performed manual actions [20]. Given that the activation of speech motor areas could affect speech categorization, there is a rationale for studying whether grasp actions could exert a similar influence. Consequently, the primary aim of the current study was to investigate whether the previously shown systematic connections between hand and mouth motor functioning [19, 20] could also work in the opposite direction, in other words if grasp actions could influence the categorization of speech sounds.

## Experiment 1

Given the connections between articulatory gestures and grip types, as well as the evidence of a tight interplay between speech production and perception, it is possible that executing a precision or power grip could also influence speech categorization. Hence, in Experiment 1 we focused on whether executing a power grip or a precision grip influenced the categorization of the syllables [ke] and [te]. We chose these syllables given previous evidence of their specific connections with these manual grips ([ke]-power, [te]-precision [19]). The speech stimuli we used were auditory, visual and audiovisual. The task was to prepare to execute a power or precision grip, then to observe a syllable being presented, to execute the prepared grip at the end of the syllable, and finally to judge whether the syllable was [ke] or [te]. There was also a no-grip condition in addition to the power and precision grip conditions, in which no grip was prepared or executed. This constituted a baseline condition, without any possible grip effects on the syllable-categorization task. The auditory stimuli were masked with pink noise in order to prevent a ceiling effect. We expected that executing a power grip would increase the proportion of [ke] responses and decrease the proportion of [te] responses, and vice versa in the case of a precision grip.

### Methods

#### Participants

There were 29 participants in the study, aged 19–37 (mean age 25.31). They were all women, right-handed, Finnish-speaking, with normal or corrected-to-normal vision, and with no reported speech, motor, hearing or neurological disorders. They gave their written consent for participation, and were given a movie ticket by way of compensation. The Ethical Review Board in the Humanities and Social and Behavioural Sciences at the University of Helsinki approved the study.

#### Equipment

**Fig 1:**
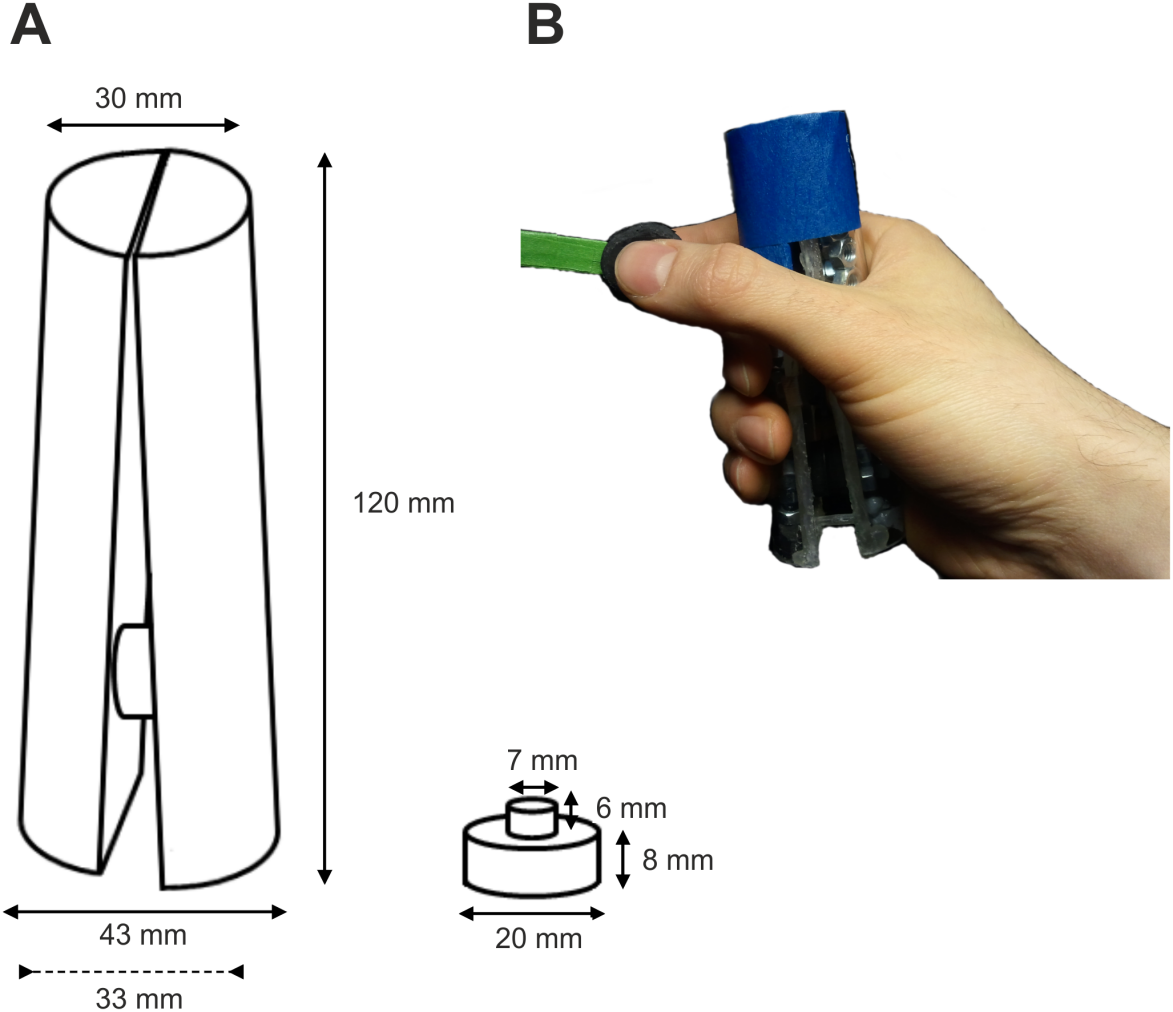
The grip devices. (A) Illustrations of the two grip devices used with their respective dimensions: the power grip device on the left and the precision grip device on the right. The dashed line under the power grip device is the minimum width of the bottom part (i.e. when the device was maximally squeezed). When the precision grip device was maximally squeezed, the cushion on top was almost completely pushed inside the bottom part of the device. The actual sensors are not included. (B) The actual devices and how the participants held them.

The participants sat in front of a monitor, holding the power and precision grip response devices in their right hand. Auditory stimuli were delivered through headphones. The two response devices were both equipped with a force-sensitive resistor (FSR 402, Interlink Electronics). Fig 1 shows the devices with the relevant size dimensions. Both devices had a short range of movement when squeezed. The precision grip device was squeezed between the thumb and the index finger, and the power grip device with the remaining fingers against the palm of the hand. The devices were marked with blue and green tape, as color cues were used to signal which grip was to be executed. The participants gave their responses to the speech-recognition task on a keyboard with the left hand.

#### Stimuli and procedure

The speech stimuli were four different iterations of a Finnish female speaker uttering the syllable [ke] or [te]. The different iterations were used to bring diversity to the stimuli as a means of preventing the participants from learning the syllables based on some non-articulation-related stimulus property. Auditory and visual stimuli were extracted from the same audiovisual video clips. The average fundamental frequency, measured from the middle of the vowel, was 230 Hz for the [ke] syllables and 238 Hz for the [te] syllables. The duration of each clip was 1800 ms. The visual utterance onset was at 480 ms from the stimulus onset (duration 490 ms), and the auditory utterance onset was at 970 ms (duration 170 ms). The face size in the trials with visual speech was approximately nine (height) and seven (width) degrees of visual angle from a viewing distance of 75 cm. The auditory speech was presented at 54 dB(A) with pink noise added to prevent a ceiling effect, and a signal-to-noise ratio of -17 dB. The selection of the noise level was based on piloting, giving a proportion of correct responses of about .75.

**Fig 2:**
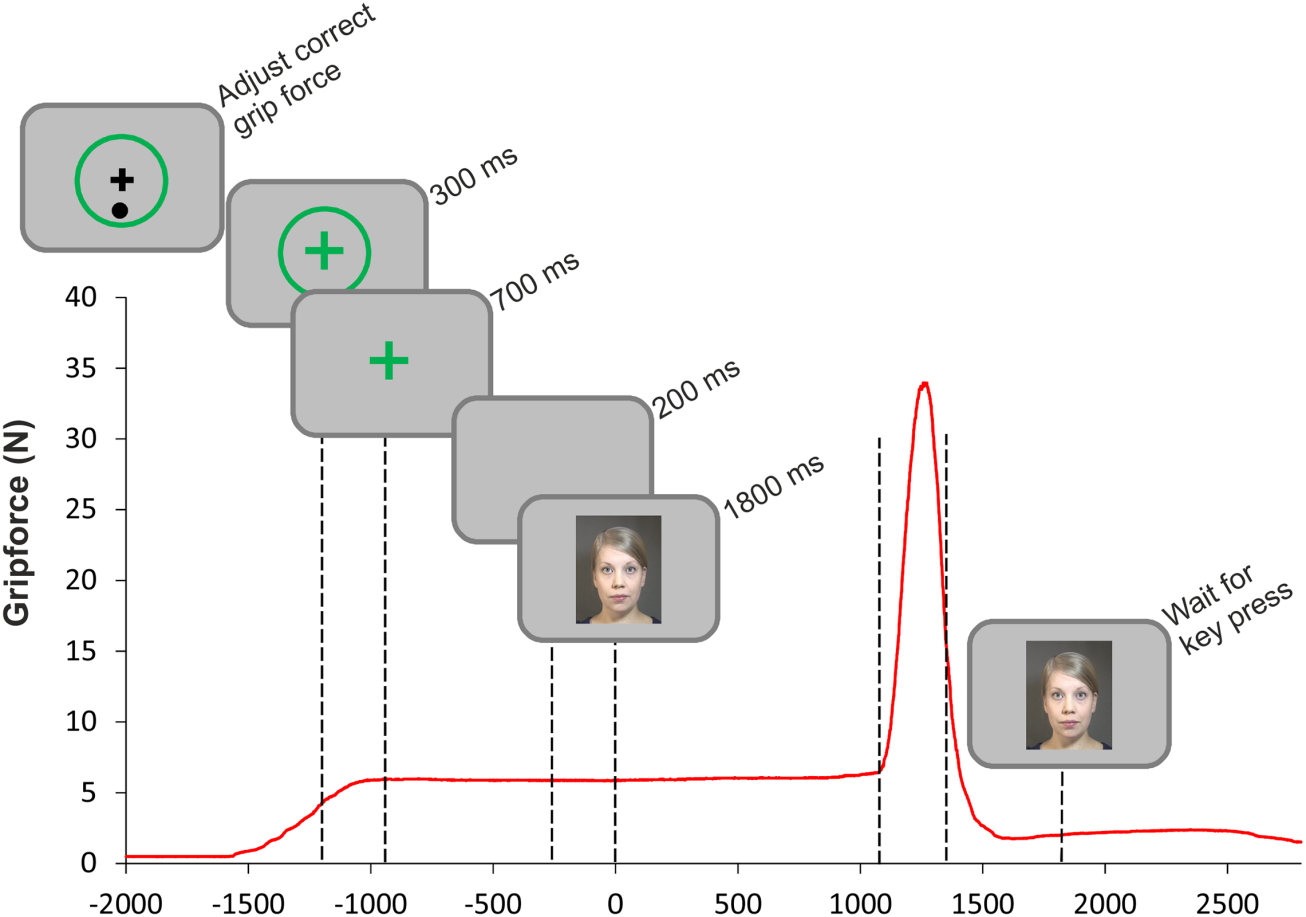
The trial structure accompanied with a graph of the associated grip-force data from an example trial. The red line charts the changes in grip force during the trial. The zero point in time is the stimulus onset. The dashed lines at around 1100 ms and 1300 ms represent the ends of the auditory and visual speech articulations, respectively. After the key press there was a 1000-ms interval before the next trial.

Each trial started with the presentation of a green or blue circle, displayed at the center of the screen. The task of the participants at the beginning of each trial was to squeeze lightly the appropriate grip device (determined by color). They were instructed to find the appropriate squeezing pressure based on onscreen instructions (see Fig 2): a black dot appeared below a black fixation cross that was presented in the center of the color circle if the grasping pressure was too weak, and above the cross if it was too strong. When the pressure was appropriate (roughly between 2.80 and 4.95 N for power and 0.68–1.20 N for precision grip), the dot and the cross were replaced with a fixation cross that had the same color as the circle. The trial did not start until the grip was properly prepared. We wanted the participants to overtly prepare the grip in order to make sure they were truly focusing on the manual grip task. The participants were required to hold that light pressure on the device while the syllable was being presented. The main manual task was to react to the end of the utterance by sharply increasing the pressure on the device that was already being slightly squeezed. After this, they responded with a left-hand key press to judge what the presented syllable was. There was no circle on the screen at the beginning the no-grip trials, just a black fixation cross, and the participants were required to refrain from squeezing the devices at any point and only to give the key-press response. Fig 2 charts the trial structure with a sample of the grip-force data.

Each stimulus combination (2 syllables × 3 grip conditions × 3 modalities) was presented 20 times (5 for each iteration of a syllable), resulting in a total of 360 trials. All the trials were presented in randomized order in a single block. Fourteen participants responded with a precision grip on the green trials and with a power grip on the blue trials, the order being reversed among the last 15 participants. Given that the task was quite difficult, each participant practiced thoroughly before the experiment proper. The experiment lasted approximately one hour and the participants had five rest breaks.

#### Data and statistical analysis

The grip data was selected from 1500 ms before to 2500 ms after the stimulus onset. Responses were labeled correct if the maximum grip force was more than 1.5 times that of the baseline (calculated from -200 to 200 ms of stimulus onset) and the correct device was squeezed harder than the incorrect one. The trials labeled as errors were manually checked to ensure that the criteria were satisfied. In the case of the no-grip trials the responses were labeled correct if the grip force did not exceed the maximum values set for the hold phase in the grip trials.

For the syllable responses we calculated the proportion of correct responses in the trials from which the erroneous grip responses had been removed. The proportions of correct responses were subjected to a repeated-measures ANOVA with the factors syllable ([ke], [te]), grip (power, precision, no grip) and modality (auditory, audiovisual, visual). Bonferroni corrected pairwise tests were carried out to further analyze the interactions.

### Results

The overall response accuracy was .77 correct for [ke] and .74 for [te]. There was a main effect of modality, *F*(2,56) = 45.52, *p* < .001, η_p_^2^ = 0.62. The correct response rate was highest (.83) for the audiovisual stimuli, second highest for the visual stimuli (.75), and lowest for the auditory stimuli (.70) (*p* < .01 in all the pairwise comparisons).

**Fig 3:**
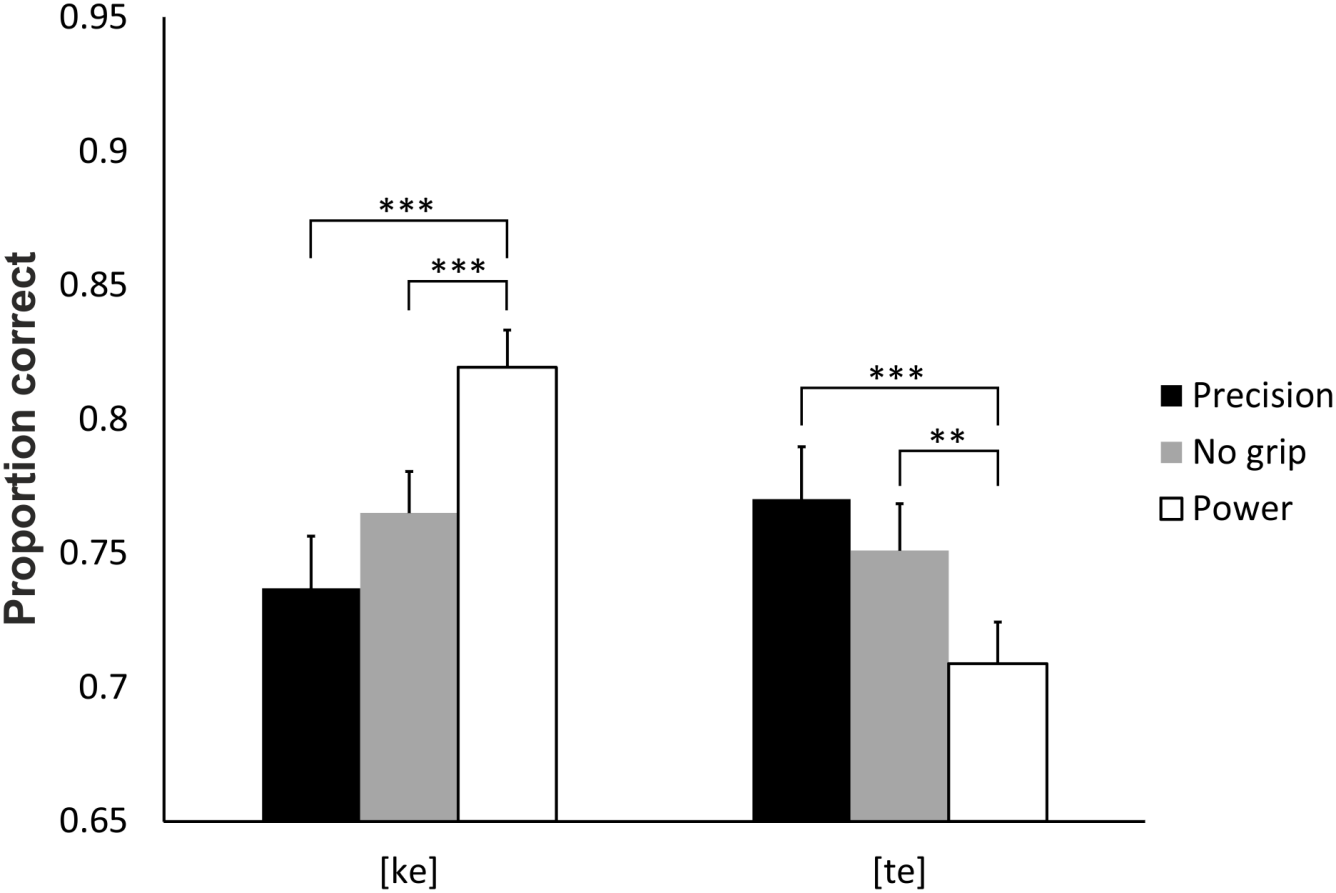
The proportions of correct responses over all modalities (auditory, visual and audiovisual) for the interaction between grip and syllable. The proportion of [ke] was higher when the participants were preparing to execute a power grip, whereas the proportion of [te] was higher when they prepared to execute a precision grip. The error bars represent standard errors. ** = *p* < .01, *** = *p* < .001.

The most important result was the significant interaction between syllable and grip, *F*(2,56) = 21.31, *p* < .001, η_p_^2^ = 0.43 (Fig 3). There were more correct responses to syllable [ke] when a power grip (.82) rather than a precision grip (.74, *p* < .001) was executed, or when there was no grip at all (.77, *p* < .001). The difference between a precision grip and no grip was not significant (*p* = .154). Conversely, there were more correct responses to syllable [te] when a precision grip (.77) rather than a power grip (.71, *p* = .001) was executed. There were more correct responses to [te] (.75, *p* = .028) in the no-grip compared to the power grip condition, and no significant difference between the precision and no-grip conditions (*p* = .623). The three-way interaction of syllable, grip and modality was non-significant (*p* = .141), indicating that this effect was replicated in all modalities.

There was also a significant interaction between grip and modality, *F*(4,112) = 3.85, *p* = .006, η_p_^2^ = 0.12. There was no difference between the auditory and the visual trials (.71 and .73, respectively, *p* = .769) with the precision grip, whereas with the power and no grip there were more correct responses in the visual (.77 and .75 respectively) than in the auditory trials (.70, *p* = .006 and .68, *p* < .001, respectively). Lastly, the interaction of modality and syllable was significant, *F*(2,56) = 58.98, *p* < .001, η_p_^2^ = 0.68: In the audio-only condition, [ke] was recognized more accurately than [te] (.86 vs. .53, *p* < .001), whereas [te] was recognized more accurately than [ke] (.86 vs. .64, *p* < .001) in the visual-only condition, and there was no difference between the syllables ([ke] = .82, [te] = .84, *p* = .647) in the audiovisual condition.

## Experiments 2 & 3

The results of Experiment 1 revealed an interaction between grip and syllable, a power grip being associated with increased [ke] responses and a precision grip with increased [te] responses. This effect could reflect the impact of grip performance on the perception of syllables, in line with similar findings that articulation influences speech-categorization tasks [7, 9, 11–13]. However, in addition to their potential influence on speech perception, actions may also modulate decision processes that are required to report a recently perceived speech stimulus. In fact, Hickok [23] challenges the interpretations of most studies claiming that activating or executing mouth movements affects the perceptual processing of speech. Instead, he posits that other mechanisms that do not necessarily involve any perception modulation could explain such results, further suggesting that the results of these studies could reflect response bias, or the tendency to systematically favor one response over another in certain situations. Indeed, according to Cisek and Kalaska’s [24] proposed framework, overlapping networks operate in the perceiving, planning and executing of actions that correspond to the task-relevant properties of stimuli, and eventually in the making of decisions about actions that are required in a task. Thus, it is theoretically plausible to assume that an action carried out in conjunction with the perceptual categorization task influences the response-selection processes that are required to report a recently perceived speech stimulus.

We therefore conducted two follow-up experiments (2 and 3). In Experiment 2 we replicated Experiment 1, but in a simplified manner. There was no need for overt grip preparation after the initial grip cue, and after the syllable ended there was a color cue to act as a go-sign for the execution. These changes made the task easier for the participants. This also allowed us to explore whether the overt grip preparation was necessary for the influence of grip on syllable categorization. We only included the audiovisual condition to increase the number of trials and thereby to utilize signal detection theory (SDT [25, 26]). The SDT analysis characterizes the participant's performance on two parameters, *d’* and *c*. Parameter *d'* (discriminability) reflects the observer's ability to discriminate between two stimuli (e.g., [ke] from [te]), whereas parameter *c* (criterion) is usually thought of as a measure of response bias, a tendency to favor one response over another (e.g., favor a [ke] response over a [te] response). Thus, SDT made it easier for us to study the role of response bias in the effect. In fact, Smalle, Rogers and Möttönen [27] used SDT in a recent TMS study addressing Hickok’s [23] criticism and found an effect in *d’* but not *c*, suggesting that the influence of articulation on speech-sound categorization is indeed perceptual.

The aim of Experiment 3 was to investigate the potential response bias more directly. In structure it was the same as Experiment 2 but without the initial cue for the grip. Because no grip was prepared beforehand, and all the grip-response information was delivered only after the syllable presentation had finished, the grip response should not have influenced the processing of the syllable. If we still observed the interaction between grip and syllable in this experiment, it would strongly support the response bias explanation.

### Material and Methods

#### Participants

Twenty-eight people (including 7 males) aged 19–50 (mean age 25.11) participated in Experiments 2 and 3. All were right-handed, Finnish-speaking, reported normal or corrected-to-normal vision and no speech, motor, hearing or neurological disorders, and all gave their written consent. They were given a movie ticket as compensation for their participation. The Ethical Review Board of the Humanities and Social and Behavioural Sciences at the University of Helsinki approved the study.

#### Equipment, stimuli and procedure

The same equipment and speech stimuli were used as in Experiment 1, except that only audiovisual stimuli were presented. Experiments 2 and 3 were conducted in the same session, however, so that what is reported here as Experiment 3 always happened first. The order was fixed to reduce potential carry-over perceptual learning effects, which one would expect to be considerably smaller from the short Experiment 3 to the long Experiment 2 than vice versa. The experiments are reported in reverse order for reasons of logic and emphasis: the main emphasis was on Experiment 2 and signal detection analysis, whereas Experiment 3 served to re-evaluate and confirm these results, as stated above.

Experiment 2 proceeded as follows. First, a fixation cross was presented for 400 ms: the cross was green, blue or black, and acted as the pre-cue for the grip that would be executed at the end of the syllable presentation (power, precision or no grip). Next, a blank screen was presented for 200 ms, after which the participants were presented with an audiovisual speech stimulus. As soon as the speaker’s mouth closed, a transparent colored circle appeared on top of the face. The circle was the go-signal for the grip response and was the same color as the pre-cue. Color mapping for the two grips was balanced between the participants, and the cue for the no-grip condition was always black. The task was to first execute the correct grip as quickly as possible and after that to report with a key press what the syllable was. There were 360 trials in total (60 for each stimulus-response combination, 2 syllables × 3 grip conditions), to provide enough data for the SDT analysis.

**Fig 4:**
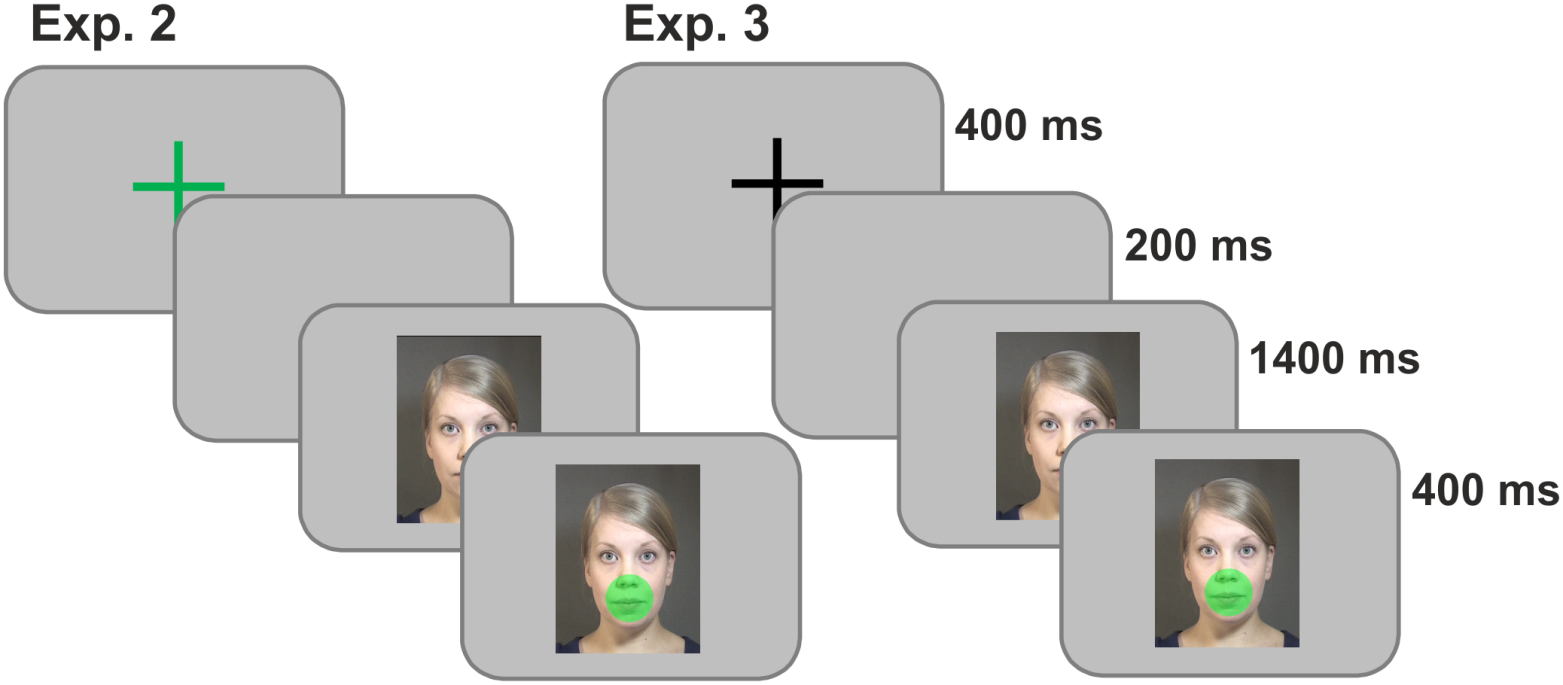
The structure of Experiments 2 (left) and 3 (right). The color of the fixation cross at the start acted as the pre-cue for the grip response in Experiment 2 and was always black in Experiment 3, otherwise the structures were identical. The fixation was presented for 400 ms, followed by a blank screen for 200 ms, after which the actual syllable began, and at 1400 ms into the syllable (when the talker had closed her mouth) the go signal for the grip was presented as a transparent circle around the speaker’s mouth.

Experiment 3 was the same in structure as Experiment 2, except that the fixation cross at the beginning of the trial was always black. Thus, the participants did not know which grip they had to execute before the speech stimulus ended. Fig 4 depicts the structure of both experiments. The number of trials in Experiment 3 was the same as the number of audiovisual trials in Experiment 1, 120 in total, making these two experiments and their results more comparable. The fewer trials also meant that Experiments 2 and 3 could fit into one measurement session.

#### Data and statistical analysis

**Fig 5:**
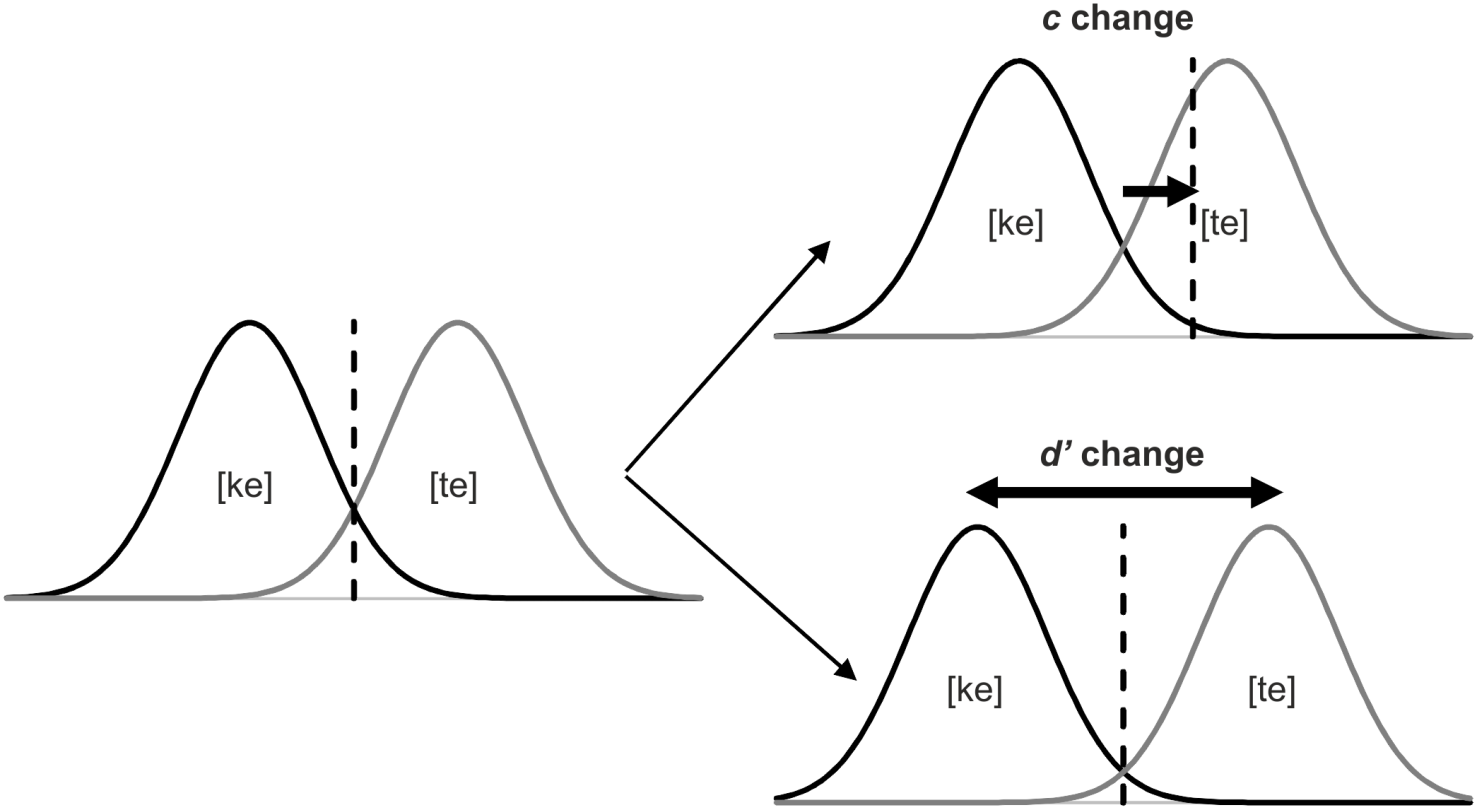
Theoretical illustrations of changes in *d’* and *c* (dashed line). The two distributions depict the representations of the two syllables. Left: when *d’ =* 3 and *c* = 0, i.e. no bias; the values to the left of the criterion indicate that the participant would respond [ke] and those to the right that he or she would respond [te]. Top right: change in the criterion to *c* = 1; almost all [ke] trials would be correctly categorized as [ke], but a large portion of [te] trials would also be falsely categorized as [ke]. Bottom right: change in discriminability to *d’* = 4; now almost all [ke] trials are correctly labeled as [ke] and almost all [te] trials are also correctly categorized as [te].

The data analysis proceeded in a similar manner as in Experiment 1. We applied signal detection theory (SDT) to further investigate the effect of grip on speech categorization in Experiment 2. SDT was originally developed to describe the detectability of signals [25], although it is also applicable to discrimination tasks [26]. Within the SDT framework the parameter *d'* usually reflects an observer's ability to discern a sensory event from its background, in other words perceptual sensitivity. In the current context we had two sensory events, [ke] and [te], instead of a sensory event and background, and *d’* was a measure of how perceptually discriminable these syllables were. The criterion parameter *c*, on the other hand, is usually thought of as a measure of response bias, the tendency to favor one response over another. Given that [ke] was chosen as the reference for the SDT analysis, a positive *c* indicated favoring a [ke] response and a negative *c* indicated favoring [te]. A value of zero would be the optimal criterion, not favoring either. In short, a modulation of *d’* with grip would mean a change in the discriminability of the syllables, and a modulation in *c* would mean a shift in the favored response [ke] or [te] (see Fig 5 for a theoretical illustration). For calculating *d’*, the situations were labeled so that hits were those in which the presented syllable was [ke] and the response was [ke], whereas false alarms (*FA*) were those in which the syllable was [te] and the response was [ke]. These values were then normalized to obtain the *z*-score values (*z*(*HIT*) and *z*(*FA*)), and subtracted from one another (i.e. *d'* = *z*(*HIT*)—*z*(*FA*)). *C* was calculated according to *c* = -0.5[*z*(*HIT*) + *z*(*FA*)]. The *d’* and *c* values could not be calculated for two participants in the no grip condition since they made no errors on the [te] syllable. Thus, they were excluded from the signal detection analysis. For the statistical analysis, the proportions of correct responses were subjected to a repeated-measures ANOVA with the factors syllable ([ke], [te]) and grip (power, precision, no grip). ANOVA’s for the *d’* and *c* included only the grip factor. Bonferroni corrected pairwise tests were carried out to further analyze the interactions.

### Results

#### Experiment 2 (response cue)

**Fig 6:**
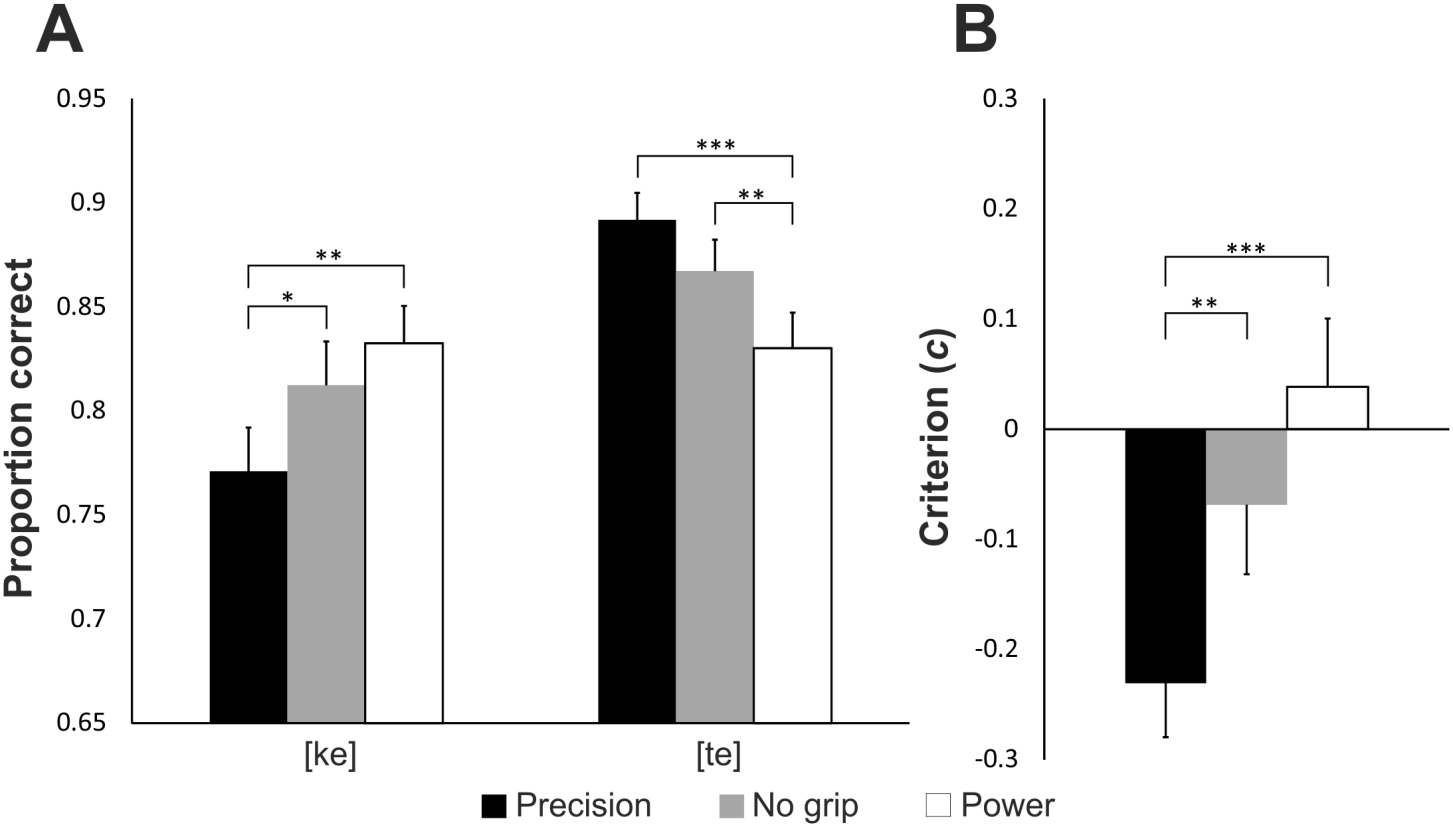
Experiment 2 results. (A) The proportions of correct responses in Experiment 2 for the interaction between grip and syllable. When the participants were prepared to execute a power grip the proportion of [ke] responses was higher, and when they were cued to execute a precision grip the proportion of [te] responses was higher. (B) The criterion values with different grips. Positive values indicate a criterion favoring a [ke] response and negative values favoring a [te] response. Zero-point is the optimal criterion with no bias. The error bars represent standard errors. * = *p* < .05, ** = *p* < .01, *** = *p* < .001.

Response accuracy was .81 for [ke] and .86 for [te]. In the ANOVA for the proportion of correct responses there was a main effect of syllable, *F*(1,27) = 5.05, *p* = .033, η_p_^2^ = 0.15, there were more correct [te] responses. The interaction between syllable and grip was also significant, *F*(2,54) = 15.23, *p* < .001, η_p_^2^ = 0.36 (Fig 6A). Pairwise analysis of the grips revealed more correct [ke] responses with a power grip (.83) than a precision grip (.77, *p* = .003), and also when there was no grip (.81) compared to a precision grip (*p* = .037). In contrast, there were more correct [te] responses with a precision (.89) than a power (.83, *p* < .001) grip, and when there was no grip (.87) compared to a power grip (*p* = .005).

Signal detection analysis revealed no significant effect of *d’* (*p* = .550, power grip *d’* = 2.06, precision *d’* = 2.08, no grip *d’* = 2.13), meaning that the discriminability of the two syllables did not change between the grip conditions. There was a criterion effect *F*(2,50) = 14.93, *p* < .001, η_p_^2^ = 0.37, favoring more [te] (vs. [ke]) responses with a precision (*c* = -0.23) as opposed to a power grip (*c* = 0.04), or when there was no grip (*c* = -0.07) (Fig 6B).

#### Experiment 3 (no response cue)

**Fig 7:**
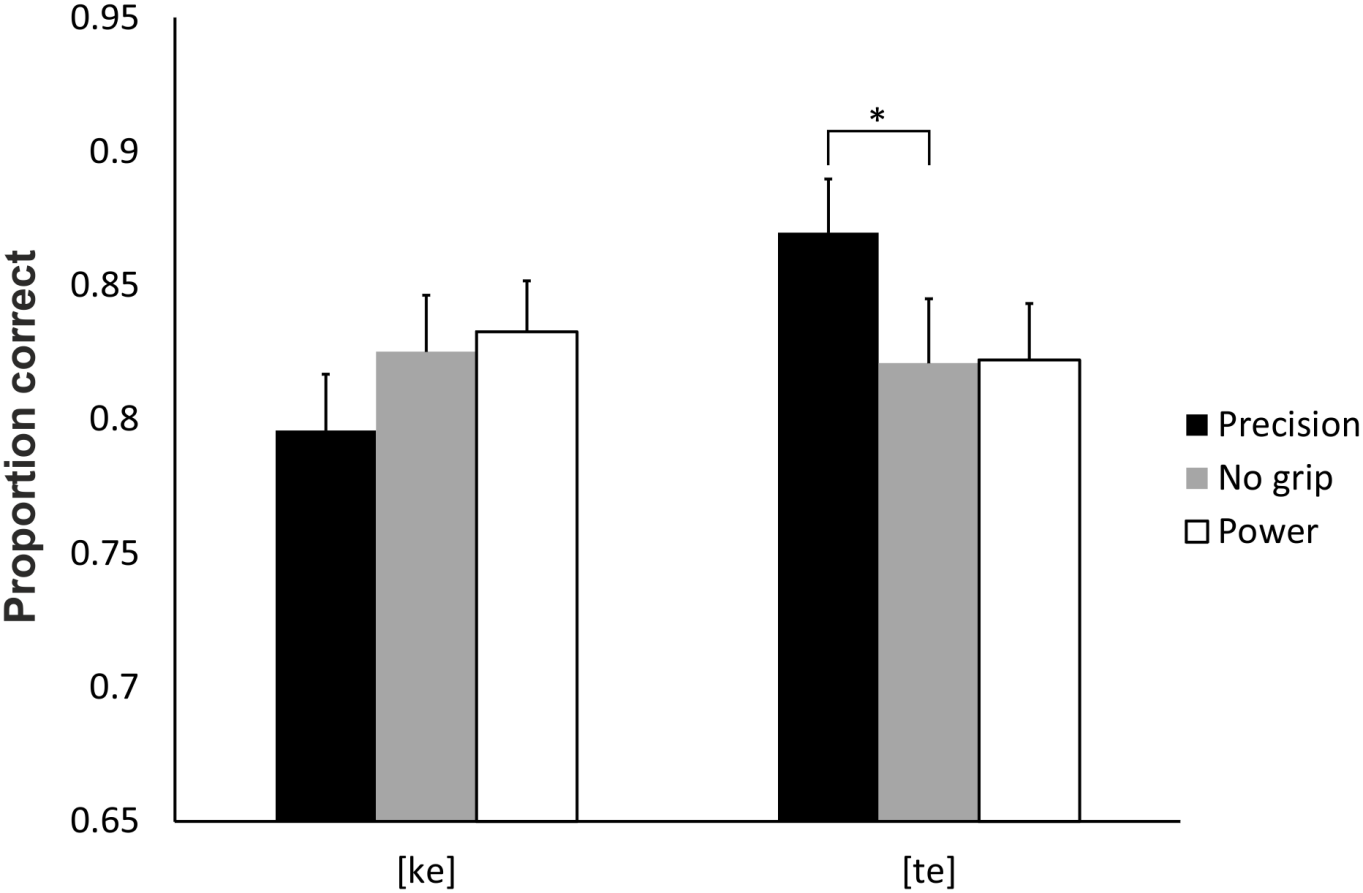
The proportions of correct responses in Experiment 3 for the interaction between grip and syllable. When the participants executed a precision grip the proportion of [te] responses was higher. The error bars represent standard errors. * = *p* < .05.

The response accuracy was .82 correct for [ke] and .84 for [te]. The only significant result of the ANOVA of the proportion of correct responses was the interaction between syllable and grip, *F*(2,54) = 4.86, *p* = .011, η_p_^2^ = 0.15. Pairwise comparison revealed that there were more correct [te] responses when a precision grip was executed as opposed to no grip. Other pairwise comparisons between grips were non-significant, but the interaction effect appeared to be similar to that observed in Experiment 2 (Fig 7), and a combined analysis of Experiments 2 and 3 revealed no difference between the two in interaction effect (syllable × grip × experiment interaction *p* = .349).

## Discussion

The results of Experiments 1, 2 and 3 revealed an influence of manual actions on syllable categorization. The participants reported more [ke] responses when executing a power grip and more [te] responses when executing a precision grip. In other words, the syllable categorization was affected by simultaneous manual grasping actions even though the actions were not directly associated with the speech stimuli. Experiment 1 showed that the effect was not dependent on stimulus modality: it was similar with auditory, visual and audiovisual stimuli. The SDT analysis of Experiment 2 revealed a criterion effect of grip, suggesting that the effect is based on criterion shifts rather than grip-modulated changes in the discriminability of the syllables. The criterion shifted to favor [ke] responses when executing a power grip, and [te] responses when executing a precision grip. The results of Experiment 3 further clarified the interpretation of these findings in revealing an effect of grip performance on syllable categorization in a situation in which grip information was presented and the grip executed after the syllable presentation. The effect was similar as in Experiment 2. This shows that at least part of the effect reflects an influence of grasp performance on selecting between response alternatives. In other words, the participants were more likely to select the syllable [te] for the response after executing the precision grip and the syllable [ke] after executing the power grip.

Cisek and Kalaska [24] suggest that all possible actions available to an individual in a given situation are processed, and the most suitable one is chosen based on biasing influences from a variety of brain systems, including sensorimotor circuits. According to this hypothesis, the same networks that are involved in carrying out an action are also heavily involved in selecting it. If the results of Experiment 3 are interpreted from the perspective of this hypothesis, executing a power grip could activate the associated networks, including the articulatory networks associated with a power grip (i.e., the articulatory representation for the syllable [ke]). Because of this increased activity in the power-grip-associated articulation network the participant would be more biased to favor this grip-compatible response, in other words to select the syllable [ke] for the response. The task used in the study enabled these action-induced biases in response selection given that the speech stimuli were unclear due to the masking of the auditory stimuli. Consequently, in a number of trials the participants were presumably required to select the syllable without being certain about what they had just perceived, opening the gate for action-induced bias in syllable selection.

These findings are somewhat similar to the results of previous research on the SNARC (spatial-numerical association of response codes) effect, where left responses are associated with low digits and right responses with high digits. For example, the presentation of a digit biases a following free-choice left-right key press [28], and when participants are required to freely pronounce a number between one and 40, larger numbers are preceded by spontaneous right and upward eye movements and smaller numbers are preceded by left and downward eye movements [29]. One interpretation of the latter finding is that a recently performed action (i.e., eye movement) influences the upcoming response selection (i.e., selecting the number for pronouncing it). The present study is the first to show that similar action-induced biases in response selection are also observable in the context of language processing, and more precisely between manual grasp actions and speech.

However, it has been recognized that in discrimination tasks, a criterion effect may also arise at the perceptual level (e.g. [30]). The location of the criterion is defined in terms of the underlying signal distributions. If the criterion stays the same on the perceptual axis, but the underlying signal representations shift equally, there is an apparent criterion shift without a change in the discriminability index *d’*. In the context of the current study, this would mean that for example under the precision grip condition, both [ke] and [te] would become perceptually more [te]-like than under the control condition, while the actual criterion stays the same. The results of Experiment 3, however, argue against this interpretation since it is not clear how the grip produced after the signal presentations could produce shifts in the signal representations.

Nevertheless, as already stated, it might be difficult to make clear distinctions between perception, action and decision-making processes [24]. Consequently, instead of trying to make final proposals as to whether the effect could reflect perceptual biases in addition to response biases, we prefer to emphasize the fact that, irrespective of the extent to which perceptual processes are involved in the effect, the underlying mechanisms are likely to be based on a common coding system between manual and oral motor actions. Gestural theories of language evolution suggest the existence of connections between hand and mouth motor functions in action-planning networks, indicating that common networks are activated when executing grasp actions and when articulating [14, 15]. Our previous study provided evidence to support this claim in showing connections between specific grasping gestures and syllable articulations [19]. A follow-up study showed that the correspondence effect is observed even when the syllable is only heard [20]. The current results extend these findings and strengthen the idea that executing manual grasps activates a partially overlapping motor network of manual and oral actions. We propose that the categorical representation of the associated syllable [ke] becomes active in the execution of a power grip, so that when a syllable is then presented the prior [ke] activation makes the presented syllable more likely to be labeled as [ke]. The converse holds for a precision grip and syllable [te].

The effect size was largest in Experiment 1, intermediate in Experiment 2 and smallest in Experiment 3. This might be due to differences in response preparation. In Experiment 1 the grip response was cued and overtly prepared beforehand. In Experiment 2 the grip was cued but not overtly prepared. In Experiment 3 no grip information was available before syllable presentation, so the grip could not be prepared. It may thus be that the stronger the grip preparation, the stronger the effect of grip on syllable categorization.

In everyday conversations, segments of speech that are not heard can be deduced from the context. Our results suggest that grip performance could also act as an implicit context cue, and according to the results of Experiment 3, also influence judgments of already processed speech. These findings provide new insight into the systematic connection between articulatory gestures and different grip types. Whether the effect is purely decision-based or also reflects perceptual bias warrants further investigation. The current study points out that the potential influence of action on the decision-making processes should be properly controlled for in future research into the influence of action on perceptual discriminability.

## Supporting Information

S1 Table Datasets used for statistical analyses. (XLSX)
